# Improved Teamwork and Implementation of Clinical Pathways in a Congenital Heart Surgery Program

**DOI:** 10.1097/pq9.0000000000000126

**Published:** 2019-01-18

**Authors:** Tina Schade Willis, Theodore Yip, Karla Brown, Scott Buck, Michael Mill

**Affiliations:** From the * Indiana University Department of Pediatrics, Division of Critical Care Medicine Indianapolis, IN; † Rutgers New Jersey Medical School Department of Physical Medicine and Rehabilitation Newark, NJ; ‡ University of North Carolina Department of Surgery, Division of Cardiothoracic Surgery Chapel Hill, NC; § University of North Carolina Department of Pediatrics, Division of Cardiology Chapel Hill, NC.

## Abstract

**Introduction:**

Complex surgical populations are at increased risk of morbidity, especially when experiencing variations in care and poor teamwork. The goal of this project was to improve teamwork and decrease variations in care in a pediatric congenital heart surgery population by implementing Integrated Clinical Pathways (ICPs) on a foundation of teamwork training.

**Methods:**

A core team used project management for completion of the project and measurement of success. The leadership team created a new operations infrastructure for the program to effectively implement and sustain improvement. Master trainers targeting teams caring for the patient population completed teamwork training and coaching. ICPs were designed and implemented using iterative tests of change with the assistance of an expert panel.

**Results:**

Three of the 4 units experienced a significant improvement in teamwork after training and coaching. The area without a significant change was one with high-level teamwork training already in place. ICPs were implemented in 2 patient subpopulations. We detected a decrease in total hours intubated using statistical process control charts in both of the ICP patient populations. Despite a decrease in intubation hours, we did not detect a reduction in length of stay in days. The infrastructure for the program was successfully implemented and remains in place 6 years later.

**Conclusions:**

Teamwork can be improved with an efficiently delivered training and coaching program. On a foundation of teamwork, ICPs can be implemented and sustained if a supporting infrastructure is in place including program leadership, buy-in from all teams, project management, and ongoing measurement.

## Abstract 2

Supplemental Digital Content is available in the text.

## INTRODUCTION

In the 17 years since the publication of the IOM report “To Err is Human,” organizations continue to struggle with improving patient safety and health.^1^ In the “Free From Harm” Expert Panel Report published 15 years after the IOM report, recommendations to improve patient safety include addressing teamwork, culture, and patient engagement.^2^ Traditionally, hospital units operate in silo fashion and have inconsistent methods of communication and care progression in and between silos.^3,4^ Poor communication is identified as a primary factor leading to error and harm especially in high-risk populations.^5–7^

The pediatric congenital heart surgery population is high-risk with wide variations in outcomes and higher mortality rates in lower volume centers.^8^ Studies have shown improved outcomes in other low-volume high-risk surgical populations after implementation of Integrated Clinical Pathways (ICPs) or teamwork training.^9,10^

One program aimed at optimizing outcomes by improving teamwork among health professionals, TeamSTEPPS^TM^, was developed by the Department of Defense and the Agency for Healthcare Research and Quality (AHRQ).^11^ Implementation of TeamSTEPPS in our institution demonstrated improved teamwork as well as decreased times to Extracorporeal Life Support cannulation.^12^ After failed attempts at standardizing rounds and implementing daily goals sheets in our pediatric intensive care unit, we successfully implemented these processes utilizing a foundation of TeamSTEPPS.^13^ ICPs (also referred to as Care Pathways and Enhanced Recovery Pathways) are evidence-based care plans that decrease variation by standardizing best practices for specific patient populations.^14,15^ ICPs are used for groups of patients with similar goals and expected LOS. It is difficult to implement and sustain ICPs even if the patient population is well defined. We theorized that implementing ICPs would be successful if we first created a foundation of improved teamwork.

### Specific Aims

1) Implement a teamwork foundation for the care of all inpatient pediatric congenital heart disease patients using a tailored TeamSTEPPS program
2) Implement ICPs for 2 common congenital heart lesions, Ventricular Septal Defect (VSD) and Tetralogy of Fallot (TOF), using evidence-based recommendations and teamwork tools

## METHODS

The target population is a medium-volume congenital heart program in an academic children’s hospital that completes approximately 180 cardiac surgical procedures annually.

A core team completed the implementation plan by using project management strategies and a charter. A multidisciplinary governance team, including family advisors, met monthly to provide guidance in the areas of personal experience, buy-in, and barriers. We referred to the overall program as “Project TICKER” (Teamwork to Improve Cardiac Kids’ End Results) with a communication platform that included a public website. The leadership team held a kick-off meeting in late 2010 with training in the first 6 months of 2011 and ICP implementation from summer 2011 through spring 2012. This project was funded under grant number R18 HS019638 from the AHRQ, U.S. Department of Health and Human Services. The project team provided a final electronic toolkit to AHRQ as required for the grant. The toolkit (available as Supplemental Digital Content at http://links.lww.com/PQ9/A58) is comprehensive and intended to assist others in adapting the program to a similar setting.

### Improvement Infrastructure and Teamwork Foundation

The structure measures included all original recommendations to the National Quality Forum for the National Voluntary Consensus Standards for Pediatric Cardiac Surgery plus the addition of teamwork training.^16^

Structure elements included the following already in place before the project:

- Participation in a multi-institutional database including volume for benchmark operations
- A multidisciplinary conference to plan surgical cases
- Availability of extracorporeal life support and transesophageal echocardiography

New elements added included:

- Multidisciplinary rounds involving all teams
- Regularly scheduled quality improvement conferences
- Teamwork training and associated communication tools

TeamSTEPPS training was tailored by mapping the process of the patient through the service line and creating specific tools. We targeted all areas of the service line for the improvement infrastructure and teamwork training even though the initial ICPs did not include patients in the Neonatal Critical Care Center. The goal was a foundation of teamwork for team members to support ICPs that would eventually target all 4 care areas:

#### Pediatric Cardiothoracic Operating Room

The operating room (OR) group consisted of 21 team members who worked in the pediatric cardiothoracic operating suites. This team had no prior TeamSTEPPS training.

#### Pediatric Intensive Care Unit

The pediatric intensive care unit (PICU) is a 20-bed unit managed by pediatric intensivists with 107 team members at the time of implementation. This team had extensive prior TeamSTEPPS training.

#### Neonatal Critical Care Center

The neonatal critical care center (NCCC) is a 58-bed unit managed by neonatologists with 194 team members at the time of implementation. This team had some prior TeamSTEPPS training.

#### Cardiac Intermediate Care Center

The cardiac intermediate care center (CICC) is an 8-bed unit offering intermediate level care to cardiac patients with 47 team members at the time of implementation. This team had no prior TeamSTEPPS training.

Additional healthcare providers chosen for TeamSTEPPS training included 89 residents, 12 pharmacists, 33 respiratory therapists, and 5 nutritionists.

Seven team members completed a 2.5-day TeamSTEPPS Master Training course and collaborated with advisory groups from each unit to implement TeamSTEPPS. All recipients of training completed a 1-hour online module. The master trainers then led 1-hour sessions for each unit over a 6-month period. In addition to the sessions, the master trainers provided in-the-moment coaching to teams during the 6-month training period. Teams utilized incremental tests of change to standardize tools for briefing, debriefing, and handoffs.

## INTEGRATED CLINICAL PATHWAY DEVELOPMENT AND IMPLEMENTATION

The project team utilized an expert panel for ICP development. The panel included representatives from cardiology, critical care, cardiothoracic surgery, nursing, anesthesiology, respiratory care, social work, and nutrition. We designed the ICPs with close consideration of all the requirements of an ICP appraisal tool and evidence-based care reviews.^15,17–19^ Frontline team members implemented the ICPs using multiple iterative pilot tests. We designed the ICPs to mimic the format of the PICU Daily Goals sheet already implemented through another quality improvement effort.^13^ Utilizing this similar format allowed the team to adopt the ICPs more rapidly due to team familiarity with the process. We implemented a similar process for utilizing pathways in the non-ICU areas.

We measured compliance during the first 4 months of implementation for each ICP. The project team collected the ICPs at the time of discharge. Compliance was successful if the paper tool was at the designated bedside location of the patient during an audit and had documentation from any care team member in both the ICU and the CICC.

The team recruited family advisors with personal experience for partnerships in the project. These advisors assisted in the development of the tools including improved family communication boards in each patient room. Photographs with names were placed next to the communication boards and updated weekly reflecting the teams that were caring for their children.

During the implementation process of ICPs, Pasquali et al.^8^ published a descriptive report of the Society for Thoracic Surgery (STS) Database describing mortality, surgical volume, and complications. They concluded that the higher mortality rate seen at low volume centers was more likely related to the recognition and treatment approach of complications than the complication rate. For this reason, expert panels designed diagnosis and treatment guidelines for some of our more common complications including venous thromboembolism, chylothorax, and junctional ectopic tachycardia.

## EVALUATION, MEASURES, AND ANALYSIS

Teamwork process measures included percent of staff trained by the end of implementation. We measured attendance to training or refresher training sessions. ICP process measures included utilization of the ICPs as assessed through elements of the ICP Assessment Tool.^17^

We evaluated teamwork as an outcome measure through observations in all 4 areas before and after implementation. Before the training periods for each unit, 5 observers used the Team Events Assessment Non-Technical Skills (TENTS) tool to evaluate teamwork.^12,20^ The TENTS tool rates 4 components of teamwork—leadership, communication, situational monitoring, and mutual support, with each component further divided into evaluated behaviors. The 4 component mean scores were averaged to give a mean teamwork score. Scores were plotted using statistical process control charts with a mean teamwork score and upper and lower control limits pre and postimplementation.^21^ We established inter-rater reliability before formally evaluating teamwork. The resulting Kappa values for the observers were in the range of 0.51–0.80 with moderate agreement.^22^

In the OR, we evaluated teamwork during the following epochs: (1) transport to OR; (2) preinduction; (3) prebypass; (4) bypass; (5) postbypass; (6) transport to ICU; (7) handoff.^23^ We also evaluated teamwork during briefings, debriefings, resuscitations, rounds, and procedural events.

Outcome measures for the ICP patients included median hospital LOS in days and total hours intubated since the complication and mortality rates were low in the ICP patients. Early in the teamwork training process, the OR and ICU teams determined that increased use of regional anesthesia, early extubation, and standardized pain and sedation goals were areas of focus for handoffs and ICPs. We followed the hospital LOS and total hours of intubation for the ICP populations through data abstracted for the STS Congenital Heart Surgery Database pre-, during, and post- ICP implementation. The total hours intubated included the operative procedure as well as any reintubation hours during the postoperative period. We followed hours intubated as recorded in the STS database to reflect the amount of time on mechanical ventilation. The baseline timeframe for outcome measurement was January 2009 through December 2010, implementation was from January 2011 through June 2012, and postimplementation was from July 2012 through June 2016. To compare the same patient populations in all time frames, we included patients if the STS surgical designation was “VSD Type 2 patch or primary repair,” or “TOF No Ventriculotomy,” “TOF Ventriculotomy,” and “TOF Ventriculotomy with Transannular Patch.” We excluded patients in the outcome measurement in all time frames if they were already hospitalized at the time of surgery since they would not be eligible for the ICP. We also excluded patients if they were intubated postoperatively for longer than 48 hours in VSD and 72 hours in TOF since they would not be eligible to remain on the pathway. The local institutional review board reviewed the project and determined that the submission was not human subjects research as defined under federal regulations.

## RESULTS

### Structure Measures

By the end of the implementation, all structure measures were in full compliance.

### Process Measures

The percent of staff TeamSTEPPS trained included 21/21 (100%) of the OR team, 94/107 (88%) of the PICU team, 170/194 (88%) of the NCCC team, 43/47 (91%) of the CICC team, 31/33 (94%) of respiratory therapists, 62/89 (70%) of residents, 4/5 (80%) of nutritionists, and 9/12 (75%) of pharmacists.

During the first 4 months of ICP implementation, there were 9 eligible VSD patients with 100% ICP compliance. During the 4 months of TOF implementation, there was only 1 eligible elective TOF patient, and the compliance was 100% for this patient. After the implementation phases of the ICPs, identification, and placement of patients on ICPs continued through a final process that is sustained 6 years after implementation. This process includes identification of ICP patients during the presurgical conference by a nurse practitioner. Eligible patients are not hospitalized preoperatively, have no significant comorbidities, and have an expected LOS of 3–5 days. Unit coordinators print pathways for these patients as they prepare the standard daily goals sheets for other patients on the morning of the patient’s surgery. The pathways follow the patient at the bedside through discharge. Patients are removed from the ICP when they have conditions that require tailored care outside of the pathway timeline.

### Outcome Measures

**Fig. 1.**
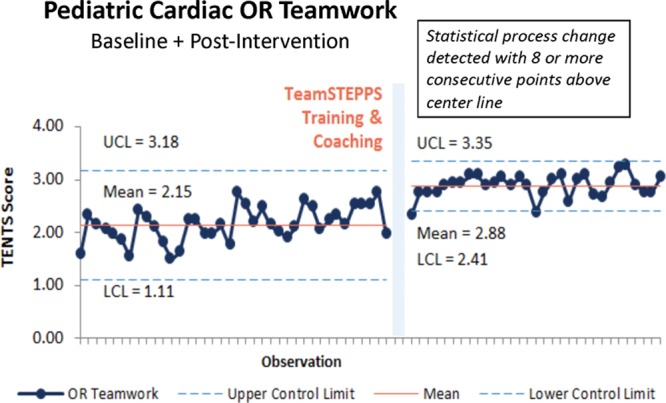
OR teamwork observation outcome measure. Baseline and postteamwork training mean TENTS score per observation as shown on a statistical process control chart with upper (UCL) and lower control limits (LCL).

Every area with new TeamSTEPPS training had improvements in teamwork scores. In the OR, we conducted 38 baseline observations and 31 posttraining observations. The pretraining teamwork mean was 2.15 and increased to 2.88 posttraining (Fig. 1).

In the PICU, we conducted 26 baseline observations and 28 posttraining observations with a mean posttraining score of 2.75. This unit did not show a process change in teamwork mean but did already have a mean score in the preimplementation period of 2.85 and had high-level training before the project.

**Fig. 2.**
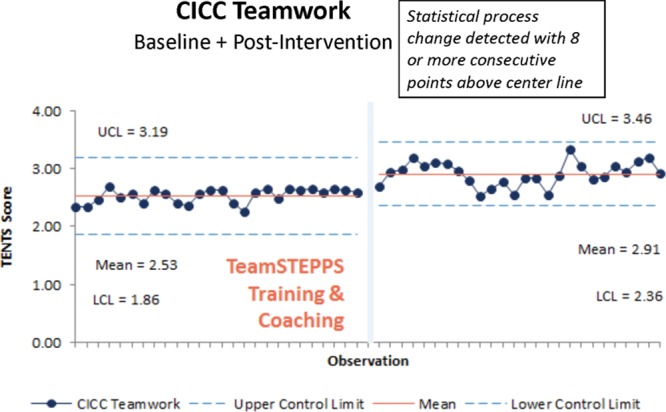
CICC teamwork observation outcome measure. Baseline and postteamwork training mean TENTS score per observation as shown on a statistical process control chart with upper (UCL) and lower control limits (LCL).

In the CICC, we recorded 26 pretraining observations with a mean baseline score of 2.53 and 26 posttraining observations with a mean posttraining score of 2.91 (Fig. 2).

**Fig. 3.**
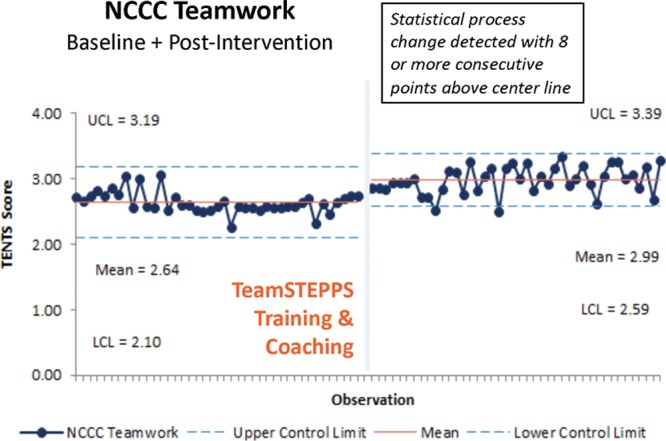
NCCC teamwork observation outcome measure. Baseline and postteamwork training mean TENTS score per observation as shown on a statistical process control chart with upper (UCL) and lower control limits (LCL).

In the NCCC, we completed 41 baseline observations with a mean prerefresher score of 2.64 and 42 posttraining observations with a mean of 2.99 (Fig. 3). Interestingly, we identified a centerline change despite the group having some exposure to TeamSTEPPS before the project.

For the VSD population, we included 85 patients in the analysis. We plotted all patients in the STS database who met inclusion criteria minus 9 patients who we excluded for postoperative intubation of longer than 48 hours. The excluded cases were 2 of 30 (7%) patients in the baseline period and 7 of 64 (11%) patients in the implementation and postimplementation period. For the TOF population, we included 39 patients in the analysis. All patients in the STS database who met inclusion criteria were plotted minus 18 patients who we excluded for postoperative intubation of longer than 72 hours. The exclusions were 4 of 13 (31%) patients in the baseline period and 14 of 44 (32%) of patients in the implementation and postimplementation periods.

**Fig. 4.**
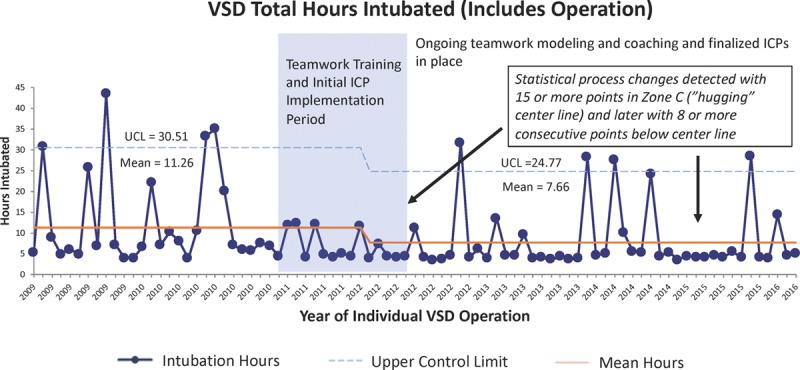
Total mean hours VSD patients intubated including the operative procedure and any postoperative reintubation hours. Data are displayed on a statistical process control chart with a center line mean and upper (UCL) and lower control limits (LCL). Patients already hospitalized at the time of surgery or requiring mechanical ventilation for greater than 48 hours were excluded since they were not eligible for initiation or continuation of the VSD ICP.

**Fig. 5.**
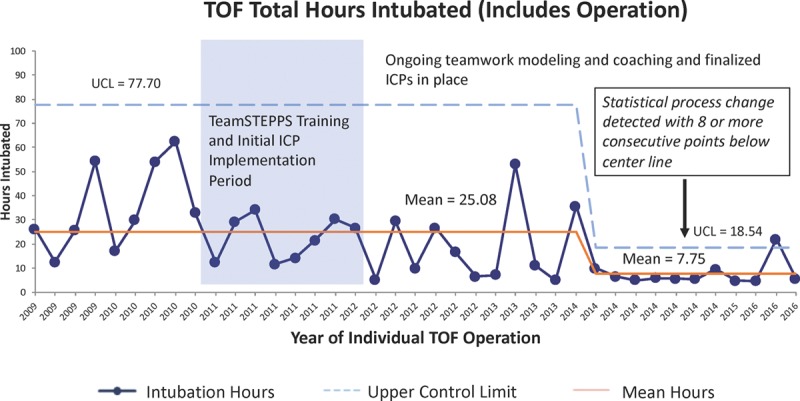
Total mean hours TOF patients intubated including the operative procedure and any postoperative reintubation hours. Data are displayed on a statistical process control chart with a center line mean and upper (UCL) and lower control limits (LCL). Patients already hospitalized at the time of surgery or requiring mechanical ventilation for greater than 72 hours were excluded since they were not eligible for initiation or continuation of the TOF ICP.

The median LOS in days for the 85 VSD patients and the 39 TOF patients remained at 5 and 7 days and did not show a statistical process control change over the preimplementation, implementation, and postimplementation periods. The total hours intubated for VSD patients decreased from a mean of 11.26 to 7.66 hours using standard statistical process control rules (Fig. 4). Similarly, the total hours intubated for TOF patients decreased from a mean of 25.08 to 7.75 hours (Fig. 5).

## DISCUSSION

Teamwork remains a key factor in providing safe care. We demonstrated that we could improve teamwork with an efficient TeamSTEPPS training program. Building upon a foundation of teamwork, all structure elements were successfully implemented and remain in place in the current program. Although TeamSTEPPS training continues with new trainees and OR teams, our institution continues to struggle with ongoing and new teamwork training. Training is completed in certain areas but not others with inconsistent refresher training and poor coordination. Training is most consistently completed when incorporated with other education such as onboarding and clinical simulation. Despite these barriers, teamwork remains a priority for our service line, and we use it as the foundation for all improvement efforts.

We have implemented ICPs, and we continue to use them for VSD and TOF populations as well as surgeries with expected hospital LOS of less than 5 days referred to as “short stay.” The institution has invested in a project manager for the program and teams continue to review and create new clinical pathways including the addition of neonatal, preoperative, and outpatient elements.

Measuring the compliance with ICPs beyond presence at the bedside is difficult since the complexity of elements in the ICP Assessment Tool is difficult to follow without project management and ICP infrastructure at the institution level. Through multiple iterative tests of change, we determined that the most effective drivers of sustainment include the program infrastructure, public access website for updated tools, leadership buy-in, and project management to pull everything together.

As one of the areas of standardization, the OR and ICU teams focused on processes that allow earlier extubation. These ICP and handoff components included the use of regional anesthesia, extubation in the OR when indicated, and standardized use of postoperative pain and sedation regimens to encourage earlier extubation in the ICU. The decrease in hours of intubation detected in both patient populations was likely influenced by many of these components and was not attributable to any one change. It was interesting that we did not detect a decrease in total hours of intubation until 2 years after initial implementation in the TOF patient population. We have continued to carry out ICP iterative tests and teamwork sustainment efforts for many years. All efforts related to early extubation are directed through the same leadership team, and we are not aware of any other initiatives that impacted this outcome measure besides ongoing teamwork and ICP efforts. Decreasing variation in care through ICPs often leads to decreased LOS. Although the LOS in days did not show a decrease as analyzed using statistical process control, the LOS in hours may have been affected. Unfortunately, this metric was not available in our STS data.

There were several limitations in our outcome measures. Since there were no ICPs in place before the project, there was no way to exclude nonpathway patients from the analysis. Instead, the same STS populations were used in all time frames and were only excluded based on the inclusion and exclusion criteria detailed. It is therefore likely that we included many patients in the pre-, implementation, and postimplementation time periods that would not have been pathway eligible. Due to various comorbidities and complications, these patients can be quite different in clinical condition and therefore not expected to have similar outcomes in time on mechanical ventilation and LOS. A quality improvement review was completed for all patients who did not remain on the pathway and for all patients with postoperative complications. Although there were several years in the project timeline, the clinical care team and senior cardiac surgeon remained consistent throughout the measurement periods.

### Lessons Learned

Partnering with family advisors helped the project team gain buy-in from all team members to prioritize and focus on new areas. For example, the idea to summarize daily goals for families in such a way that empowers them to be proactive in their child’s recovery came directly from discussions with advisors. Frontline team members reported that they were more likely to participate in the project because of the engagement of family advisors.

This project was approved for the quality improvement portion of Maintenance of Certification through the American Board of Pediatrics and was an incentive for participation. Physicians were required to complete TeamSTEPPS training and project activities such as attending meetings, piloting tools, and submitting feedback.

The addition of a quality improvement conference with institution-specific data scheduled adjacent to the surgical planning conference was well received. This intervention led to a permanent process for targeted improvement work led by smaller multidisciplinary teams.

## DISCLOSURE

The authors have no financial interest to declare in relation to the content of this article.

## Supplementary Material
